# Grafting with RAFT—gRAFT Strategies to Prepare Hybrid Nanocarriers with Core-shell Architecture

**DOI:** 10.3390/polym12102175

**Published:** 2020-09-23

**Authors:** José L. M. Gonçalves, Edgar J. Castanheira, Sérgio P. C. Alves, Carlos Baleizão, José Paulo Farinha

**Affiliations:** Centro de Química Estrutural and Department of Chemical Engineering, Instituto Superior Técnico, University of Lisbon, 1049-001 Lisboa, Portugal; joseluis@tecnico.ulisboa.pt (J.L.M.G.); e.castanheira@ua.pt (E.J.C.); sergio.c.alves@tecnico.ulisboa.pt (S.P.C.A.)

**Keywords:** RAFT polymerization, grafting strategies, smart nanocarriers, core-shell hybrid mesoporous silica nanoparticles, pH-responsive polymeric shell

## Abstract

Stimuli-responsive polymer materials are used in smart nanocarriers to provide the stimuli-actuated mechanical and chemical changes that modulate cargo delivery. To take full advantage of the potential of stimuli-responsive polymers for controlled delivery applications, these have been grafted to the surface of mesoporous silica particles (MSNs), which are mechanically robust, have very large surface areas and available pore volumes, uniform and tunable pore sizes and a large diversity of surface functionalization options. Here, we explore the impact of different RAFT-based grafting strategies on the amount of a pH-responsive polymer incorporated in the shell of MSNs. Using a “grafting to” (gRAFT-*to*) approach we studied the effect of polymer chain size on the amount of polymer in the shell. This was compared with the results obtained with a “grafting from” (gRAFT-*from*) approach, which yield slightly better polymer incorporation values. These two traditional grafting methods yield relatively limited amounts of polymer incorporation, due to steric hindrance between free chains in “grafting to” and to termination reactions between growing chains in “grafting from.” To increase the amount of polymer in the nanocarrier shell, we developed two strategies to improve the “grafting from” process. In the first, we added a cross-linking agent (gRAFT-*cross*) to limit the mobility of the growing polymer and thus decrease termination reactions at the MSN surface. On the second, we tested a hybrid grafting process (gRAFT-*hybrid*) where we added MSNs functionalized with chain transfer agent to the reaction media containing monomer and growing free polymer chains. Our results show that both modifications yield a significative increase in the amount of grafted polymer.

## 1. Introduction

The development of smart polymer materials has been inspired by the response to stimuli present in all living organisms. These materials are designed to recognize a specific environmental stimulus and respond to it in a specific way, generally with the material returning to the original state in the absence of the stimulus [1,2]. One promising application of smart polymer materials is in the control of cargo delivery at the nanoscale, ranging from drug delivery to catalysis, corrosion control and so forth [3,4,5].

Stimuli-response in polymers materials is generally related to changes in the polymer chain conformation [6] that can be induced by temperature [7,8], pH [9], light [10], proteins [11], ionic strength [12], and so forth or combinations thereof [13,14]. Among these stimuli, pH has many potential applications because it can be used as an intrinsic trigger in processes where variations in pH occur as a consequence of changes that need a particular response. For example, metal scavenging for environmental remediation [15], fertilizer regulation in agriculture [16,17], corrosion protection [18], drug delivery [19], and so forth. The conformation change of the polymer can be tuned to the desired application by selecting the acid or basic monomer building blocks [20,21].

Although smart polymers can be used in controlled delivery systems such as micro-gels and capsules, these systems generally present poor mechanical properties and low cargo capacity. One way to overcome these limitations is to combine the stimuli-responsive polymers with another support to create hybrid nanocarriers, where the polymers grafted at the surface of the support are used to modulate cargo release [1,2,22,23]. Mesoporous Silica Nanoparticles (MSNs) have been shown to offer several design advantages as a support for this application, including high cargo loading capacity (available pore volume > 1 mL/g), high mechanical and chemical stability, well-defined particle morphology, tunable pore geometry and diameter (ordered/worm-like; 2–8 nm), a wide range of particle diameters (from 15 nm to several hundred nanometers), good colloidal stability and biocompatibility and the possibility to independently functionalize their inner (pore) and outer surfaces [24,25,26].

To develop smart hybrid nanocarriers where a shell of stimuli-responsive polymer controls the release of cargo from a MSN core, it is of paramount importance to have a precise control over the length and architecture of the polymer chains to achieve a reproducible, reliable and uniform stimuli-response. Reversible addition fragmentation chain transfer (RAFT) polymerization mimics the control over polymer molar mass dispersity, composition and architecture offered by living polymerization [27], but provides better design flexibility because it requires milder experimental conditions and works for a wide range of monomers [28,29,30,31,32]. It also offers high end-group fidelity, allowing convenient and reliable grafting options to prepare hybrid nanocarriers [1,2,22,33].

The combination of RAFT polymerization with graft approaches (gRAFT) to modify the surface of nanomaterials can follow one of the common “grafting to” or “grafting from” approaches. In “grafting to” (gRAFT-*to*), pre-formed polymer chains are anchored to the surface using the chain end-group, while in “grafting from” (gRAFT-*from*) the polymer chains grow from anchoring points (usually the RAFT chain transfer agent, CTA) at the surface. 

Here, we explore and modify these two main processes to optimize the grafting of a RAFT pH-responsive polymer, poly(2–(diisopropylamino) ethyl methacrylate), to the surface of MSN nanocontainers. Our goal is to understand how the architecture (linear chains or gel-like) and design (chain size and chain size distribution) of the polymer shell influences polymer incorporation and, therefore, the cargo delivery control performance of the system. Starting with the gRAFT-*to* approach, we determined the impact of the polymer chain size on the amount of polymer incorporated in the MSN shell. We then compare the influence of the monomer concentration and ratio of initiator to CTA on the performance of the gRAFT-*from* approach. Finally, we optimize the gRAFT-*from* procedure to incorporate the polymer shell by taking advantage of the more malleable character of the “grafting-from” approach and introducing either a cross-linking agent (gRAFT-*cross*) or combining it with free-growing polymer chains in a hybrid grafting approach (gRAFT-*hybrid*).

## 2. Materials and Methods

### 2.1. Materials and Reagents

N-cetyltrimethylammonium bromide (CTAB, 99% Sigma-Aldrich, St. Louis, MO, USA), absolute Ethanol (99.9% EtOH, Scharlab, Barcelona, Spain), hydrochloric acid (HCl, 37%, VWR, Lutterworth, England), Ammonium Hydroxide (NH_4_OH, 28%, Sigma-Aldrich), tetraethoxysilane (TEOS, 99%, Sigma-Aldrich), 3-aminopropyl triethoxysilane (APTES, 98%, Sigma-Aldrich), sodium hydroxide (NaOH, Nouryon, Barcelona, Spain), *N*-trimethoxysilylpropyl-*N, N, N*– trimethylammonium chloride (CAT, 50% ethanol, Gelest, Morrisville, PA, USA), 4-(Dimethylamino) pyridine (DMAP, 98%, Sigma-Aldrich), *N*-(3-Dimethylaminopropyl)-*N′*-ethylcarbodiimide (EDC, 98%, Sigma-Aldrich), ethylene glycol dimethacrylate (EGDMA, 98%, Sigma-Aldrich) and 4-Cyano-4-(phenylcarbonothioylthio)pentanoic acid (CPADB, 97%, Sigma-Aldrich) were used as received. Azobisisobutyronitrile (AIBN, 99%, Sigma-Aldrich) was recrystallized from trifluoroacetic acid (>99%, Merck) and 1,4-dioxane (>99.9 %, Sigma-Aldrich), acetonitrile (spectroscopic grade, Panreac, Barcelona, Spain) and absolute ethanol (99.9% EtOH, Scharlab) were used as received. Toluene, triethylamine and dichloromethane were distilled over calcium hydride prior to use. Tetrahydrofuran (99% THF, Sigma-Aldrich) was distilled over sodium, prior to use. The monomer 2–(diisopropylamino) ethyl methacrylate (DAEM) was synthesized according to the literature [34]. Deionized water from a Millipore (Millipore: Burlington, MA, USA) Milli-Q ≥ 18 MΩ cm system (with a Millipak membrane filter 0.22 µm) was used in the preparation of solutions and in synthesis.

### 2.2. Synthesis of Mesoporous Silica Nanoparticles (MSNs) 

MSNs were synthesized following a method previously reported [24]. In a 500 mL polypropylene flask, deionized (DI) water (240 mL), CTAB (0.500 g) and a NaOH solution (1.7 M, 1.75 mL) were added. For PDI-labeled MSNs, the solid mixture CTAB/PDI was added to the flask at 32 °C. Afterwards, TEOS (2.5 mL) was added dropwise and the solution was left stirring for 3 h. The particles were recovered by centrifugation and the solid was washed with a mixture of ethanol and water. The solid (MSNs-CTAB) was dried at 50 °C overnight and latter under vacuum.

### 2.3. MSNs Amine Functionalization (MSN-NH_2_)

The MSNs containing the template (MSN-CTAB, 0.6 g) were dispersed in a solution of APTES (0.15 mL) in dry toluene (22 mL). The reaction mixture was kept at 130 °C under argon for 24 h. The nanoparticles were recovered by centrifugation and washed with ethanol. For template removal, the nanoparticles were re-suspended in an acidic ethanol solution ([HCl] = 0.5 M, 25 mL per 500 mg of particles) and sonicated for 15 min. The mixture was left under stirring at 50 °C for 24 h and the nanoparticles were recovered by centrifugation and washed with a basic ethanol solution (NH_4_OH:ethanol, 3:1). The solid (MSN-NH_2_) was dried at 50 °C for 24 h. 

### 2.4. Surface Modification with a RAFT Chain Transfer Agent (MSN-CTA)

MSN-NH_2_ were dispersed in dry dichloromethane (23 mL) under argon atmosphere and sonicated for 20 min. Then, EDC (1.2 eq. to APTES) and the chain transfer agent CPADB (1 eq. to APTES) were added to the mixture and left stirring at room temperature for 24 h. MSN-CTA were recovered by centrifugation and washed with ethanol. The product (MSN-CTA) was dried at 50 °C for 24 h. The amount of RAFT agent at the MSNs surface was calculated by UV-Vis spectroscopy using the molar extinction coefficient of the RAFT [35].

### 2.5. RAFT Polymerization of DAEM in Solution.

In a vial, DPA (1 eq.), TFA (1.25 eq.) and 0.5 mL of absolute EtOH were stirred for 10 min. After, AIBN initiator and CPADB were added separately with EtOH ([AIBN]:[CPADB] = 1:5). The AIBN solution was first transferred to the vial with CTA and the resulting mixture transferred to the vial containing the monomer. In each transfer the vials were washed with EtOH. Finally, the mixture was transferred to a Schlenk tube and left under argon atmosphere. The total volume of EtOH was 1.8 mL. The Schlenk was submitted to five freeze pump cycles. The mixture was then placed in an oil bath at 80 °C for 24 h. The polymer was precipitated with diethyl ether and the Schlenk was washed to remove residues. Then, the polymer was dissolved in ethanol and evaporated until dryness. Finally, it was dried under vacuum and a solid product was obtained. We prepared two samples of pDAEM with different sizes, by changing the [DAEM]:[CTA] ratio (235:1 for pDAEM47 and 47:1 for pDAEM15). The conversion of the monomer was monitored by taking samples in intervals of 15 min during the first hour and 30 min for the next 3 h (Figure S1, Supporting Information). The reaction’s conversion was determined by ^1^H NMR using 1,3,5-trioxane as the internal standard (Figure S2, Supporting Information).

### 2.6. Polymer Grafting at the MSN Surface (MSN-pDAEM)

The preparation of MSN-pDAEM hybrid nanocarriers was carried out using three different methods to obtain the polymer shell.

#### 2.6.1. Grafting to Approach, gRAFT-to

In a round flask, MSN-NH_2_ (50 mg, 1.5 mmol of APTES per gram of MSN) were dispersed in acetonitrile (2 mL) and sonicated for 30 min under argon atmosphere. To the particle suspension, a solution of DMAP (0.2 eq.), EDC.HCl (1.5 eq.) and the pDAEM (0.1 eq.) (see Section 2.5) in acetonitrile (1 mL) was added. The reaction proceeded for 24 h. The particles were recovered by centrifugation and washed three times with ethanol. The particles (MSN-pDAEM-*to*) were dried at 50 °C overnight and later under vacuum.

#### 2.6.2. Grafting from Approach, gRAFT-from

In a Schlenk flask (A), MSN-CTA nanoparticles (40 mg, 0.08 mmol of CTA per gram of MSN) were added under argon atmosphere. In another Schlenk (B), DAEM monomer (1 eq.), TFA (1.5 eq.), AIBN (adjusted for the target [AIBN]/[CTA] ratio) and ethanol (adjusted for the target DAEM concentration) were added under argon atmosphere. The flask was submitted to five freeze pump cycles. The mixture was transferred from (B) to (A) with a cannula and then place in an oil bath at 80 °C for 24 h. The core–shell nanoparticles (MSN-pDAEM-*from*) were recovered by centrifugation and washed with ethanol. The particles were dried at 50 °C overnight and later under vacuum. The crosslinked MSN-pDAEM (MSN-cross-pDAEM-*from*) were prepared using the same procedure but adding EGDMA (0.04 eq.) to Schlenk (B) prior to the freeze-pumping procedure.

#### 2.6.3. Hybrid Grafting Approach, gRAFT-hybrid

In a Schlenk flask (A), MSN-CTA nanoparticles (100 mg, 0.08 mmol of CTA per gram of MSN) and AIBN (0.27 mg) were added under argon atmosphere ([AIBN]:[CTA] = 1:5). In another Schlenk flask (B), a polymerization reaction (check Section 2.5) was conducted using two times the quantity of CTA present in Schlenk A ([AIBN]:[CTA] = 1:5). The monomer concentration was 1.3 M. When the polymerization in Schlenk B reached ca. 50% conversion (see Figure S1, Supporting Information) the mixture from Schlenk B was transferred to Schlenk A with a cannula under argon and left at 80 °C for 24 h. We run two polymerizations, with different [DAEM]:[CTA] ratio (47:1 for MSN-pDAEM-hybrid-1 and 235:1 for MSN-pDAEM-*hybrid*-2). The core–shell nanoparticles (MSN-pDAEM-*hybrid*) were recovered by centrifugation and washed three times with ethanol. The particles were dried at 50 °C overnight and later under vacuum.

### 2.7. Methods

Transmission Electronic Microscopy (TEM, Hitachi High-Technologies, Tokyo, Japan), model H-8100, with a LaB6 filament (Hitachi High-Technologies Europe GmbH, Krefeld, Germany) complemented with an accelerator voltage of 200 kV and a current of 20 µA. A camera KeenView (Soft Imaging System, Münster, Germany) is incorporated in this equipment, which, through iTEM software, allows the acquiring of TEM images. MSN dispersed in ethanol were prepared and dried on a Formvar carbon coated copper grid 200 mesh (Ted Pella, Redding, CA, USA). 

Zeta-potential–Zetasizer Nano ZS (Malvern, Malvern, United Kingdom), model ZEN3600. Zeta potentials were calculated from electrophoretic mobility using the Smoluchowski relationship. Disposable folded capillary cells (DTS1070) were used for the measurement of zeta potentials. All measurements were performed in triplicate. 

Size Exclusion Chromatography with Multi Angle Light Scattering detection (SEC-MALS, Shimadzu, Kyoto, Japan) Prominence system, consisting of a LC-20AD peristaltic pump, a DGU-20A3R degassing unit, a CTO-20AC columns temperature oven and a Rheodyne 7725i injector (injection volume of 50 µL). Three detectors in series were used—a Shimadzu Prominence RF-20A fluorimetric detector, a multiangle static light-scattering (MALS) Wyatt (Wyatt, Santa Barbara, CA, USA) MiniDawn Treos detector and a Shimadzu RID-10A Refractive Index detector (internal temperature 40.0 °C). The chromatography column was one Phenolgel analytical column (30 cm × 7.8 mm, pore sizes of 10^3^ Å; column temperature: 23.0 °C, Phenomenex, Torrance, CA, USA) with guard cartridge, using dry THF as the eluent at a flow rate of 0.8 mL/min.

^1^H NMR: Bruker AMX-400 instrument (Bruker, MA, USA). UV–Vis spectroscopy: UV-660 UV–VIS Spectrophotometer (JASCO International, Tokyo, Japan), supplied with a double monochromator and a photomultiplier detector for higher resolution. To quantify the polymer incorporated in the MSNs by ^1^H-NMR, two solutions of NaOH (pH 13) and 1,3,5-trioxane in D_2_O were prepared. The NaOH destroys the silica leaving the polymer intact and the 1,3-dioxane acts as internal standard. In a NMR tube, 5.0 mg of particles, 400 μL of NaOH solution and 100 μL of 1,3,5-trioxane solution were mixed and sonicated, until a clear solution was obtained.

## 3. Results and Discussion

**Figure 1:**
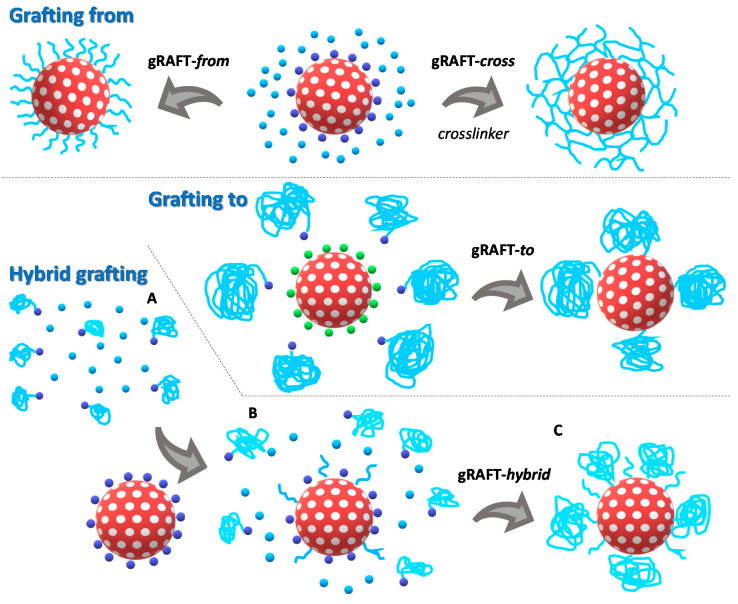
Grafting strategies used to incorporate a pH-responsive polymer on the surface of mesoporous silica nanoparticles (MSNs). In the “grafting from” approach (gRAFT-*from*) the monomer 2–(diisopropylamino) ethyl methacrylate (DAEM, **light blue spheres**) polymerization is mediated by the CTA (**dark blue spheres**) anchored at the surface of the MSNs (**porous red spheres**), therefore, the polymer chains (**light blue lines**) grow anchored to the MSN surface. When the same strategy is performed in the presence of a crosslinker (gRAFT-*cross*), a polymer gel shell is obtained. In the “grafting to” approach (gRAFT-*to*), previously synthesized RAFT polymer chains (**light blue lines**) with a carboxylic acid end-group (**blue sphere**) are grafted to the surface of MSNs, by reaction between the chain end and the primary amine groups decorating the MSN surface (**green spheres**). In the “hybrid grafting” approach (gRAFT-*hybrid*) the polymerization is initiated in solution in the absence of MSNs (**A**). When the polymerization conversion reaches ca. 50%, the MSNs (**porous red spheres**) functionalized with CTA (**dark blue spheres**) are added to the reactional medium containing the monomer (DAEM, **light blue spheres**) and the growing polymer chains (**light entangled blue lines**) and the reaction proceeds for 24 h (**B**). During this time, there are free chains and chains anchored to MSNs chains simultaneously growing, with termination reactions between free and grafted chains occurring to produce a polymer shell with a broad chain size distribution (**C**).

The preparation of a polymeric shell (and its corresponding density) on nanoparticles is influenced by several factors—the polymer molecular weight, the presence of a cross-linking agent, the anchoring strategy and so forth. To understand how the structure of the polymer shell influences the polymer incorporation at the surface of nanoparticles, we used RAFT polymerization to accurately design our pH-responsive shell in terms of size and architecture. We combined the control provided by RAFT polymerization with the versatility offered by different grafting processes (Figure 1). 

The “grafting from” (gRAFT-*from*) approach, where the polymer chains grow from surface-anchored RAFT chain transfer agent (CTA), provide high surface coverage but relatively short polymer chains due to generally large chain termination rates related to radical proximity. On the other hand, the “grafting to” (gRAFT-*to*) approach, where polymer chains pre-formed by RAFT are anchored to the surface using reactive chain-end groups, provides excellent control over the size, composition and architecture of the polymer chains. Nevertheless, steric hindrance between anchored and approaching polymer chains and the low availability of the chain end-group (both phenomena become more prevalent as the polymer chain size increases) could result in a low coverage. To increase polymer incorporation in the “grafting from” approach, one can try to reduce the termination rate by introducing a cross-linking agent to reduce chain mobility, in a crosslink-grafting from approach (gRAFT-*cross*). Another strategy is to use a “hybrid grafting” approach (gRAFT-*hybrid*) where both monomer and free-growing RAFT polymer chains are present when CTA-functionalized MSNs are introduced in the reaction mixture. In this “hybrid grafting” strategy both pre-formed chains and surface anchored chains grow simultaneously, contributing to a larger polymer density at the surface of the nanoparticles, although at the cost of a broader chain size distribution.

**Figure 2:**
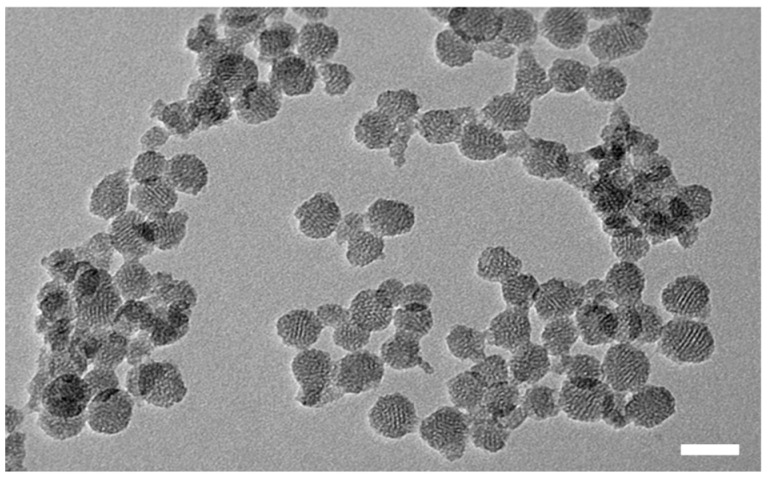
TEM image of the MSNs with the pore structure. Scale bar = 50 nm.

Our pH-actuated hybrid nanocarriers use the versatility and high cargo loading capacity of mesoporous silica nanoparticles (MSNs) [24,26]. The external surface of the MSNs can be functionalized to incorporate the pH-responsive polymer shell, while maintaining the pores free to load and carry cargo [1,2,22,23]. The MSNs were first prepared by an aqueous low temperature sol-gel process that allows full control over their size and pore morphology [24,36]. The MSNs have an average diameter of (45 ± 9) nm (obtained from the analysis of 50 particles in TEM images) and feature a pore structure that is visible in the TEM image (Figure 2). 

**Figure 3:**
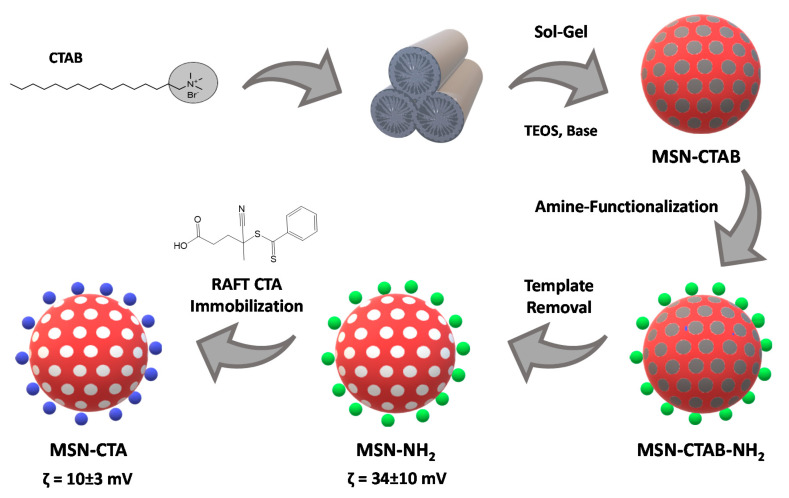
Schematic illustration of the preparation of functionalized MSNs. Zeta-potential values measured at pH = 5.6 reflect the change in surface charge upon surface modification.

The MSNs were surface modified with APTES to incorporate amine groups on their external surface. To prevent modification of the pore surface, this procedure was performed before template removal. The amine concentration at the MSN surface was 0.84 mmol·g^-1^ (determined by ^1^H NMR) [37], which corresponds to a surface coverage of 1.7 amine groups per nm^2^ (Figure S3, Supporting Information). After removing the template, the CTA agent was covalently linked to the MSNs by reaction with the amine groups in the presence of EDC. The amount of immobilized CTA was 0.08 mmol·g^-1^ (determined by UV-Vis spectroscopy) [33], corresponding to a surface density of 0.16 molecules per nm^2^ (Figure S4, Supporting Information). The success of each step was confirmed by zeta-potential measurements (Figure 3), with the change in zeta potential upon immobilization indicating a clear change on the nanoparticles external surface charge.

### 3.1. Grafting-to Approach, gRAFT-to

In the “grafting to” approach (gRAFT-*to*), the polymer chains are obtained by RAFT with pre-determined size, composition and architecture. The polymer can thus be easily characterized before anchoring to the nanocarrier so that the characteristics of the obtained polymer shell are well known.

We used RAFT polymerization to prepare pDAEM of different molecular weight by changing the monomer to CTA ratio, with a constant initiator:CTA ratio of 1:5. The molecular weight (*M*_n_) and size dispersity of the polymer were determined by SEC-MALS (Figure S5, Supporting Information) as *M*_n_ = 47 kDa for pDAEM47 and *M*_n_ = 15 kDA for pDAEM15, both with low dispersity (*M*_w_/*M*_n_ = 1.04). These values are close to those expected from the monomer feed for 87.5% conversion (Table 1; see Figure S1, Supporting Information) 

The chain end-group is then used to anchor the polymer to the surface of the amine-modified nanocarrier, MSN-NH_2_, in the presence of ethylcarbodiimide (EDC) and 4-(Dimethylamino) pyridine (DMAP). The polymer incorporation was determined by ^1^H NMR using trioxane as internal standard, after destroying the silica core at basic pH (Table 1) [37]. The amount of polymer incorporated in the MSNs was 1.0 wt % for MSN-pDAEM47-*to* and 0.5 wt % for MSN-pDAEM15-*to*, corresponding to an average of 7.1 and 10.4 chains per MSN, respectively. The surface grafting density values are limited by both the steric hindrance between polymer chains and the low availability of the chain end-group to react at the MSN surface. On one hand, it becomes increasingly difficult for the incoming polymer chains to approach the surface due to the steric hindrance imposed by the chains previously grafted [38,39]. On the other hand, the grafting kinetics is slowed down by the random coil conformation which obstructs access of the reactive chain-end group to the surface. The higher polymer incorporation obtained for pDAEM47 can be attributed to the higher molecular weight of this polymer, which compensates for the lower average number of chains per MSN.

### 3.2. Grafting from Approach, gRAFT-from

**Figure 4:**
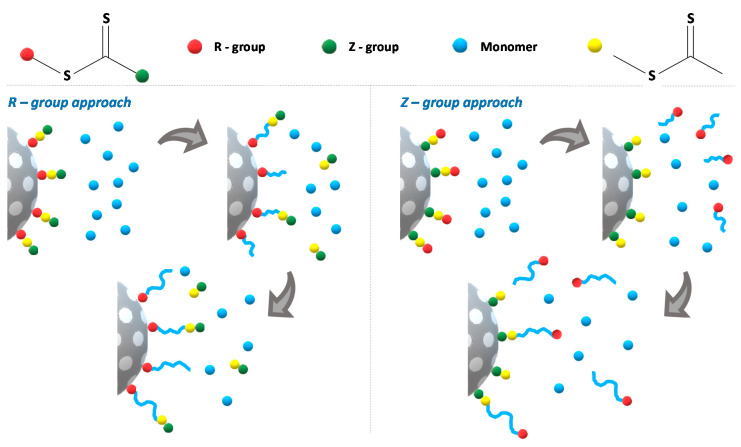
Strategies for surface-initiated RAFT polymerization (gRAFT-from) using a dithioester CTA (equivalent for a trithiocarbonate CTA). In the R-group approach, the RAFT is immobilized by the R group and the grafted polymer chains grow from the surface with the propagating radicals easily accessible on the terminal end for the chain-transfer reactions. In the Z-group approach, the RAFT is immobilized by the Z group, the polymer chains grow in solution and the chain transfer reactions occur near the surface of the material, hampered by steric hindrance from the neighboring attached polymer chains and by the low availability of the chain end-groups.

Surface-initiated RAFT polymerization (gRAFT-*from*), where the polymer chains grow from surface-anchored CTA, has been widely used to achieve high grafting densities with precise control over the size and structure of the grafted polymer chains. We used the R-group approach [30], with the CTA attached to the MSNs external surface by reaction of the R group (the carboxylic acid group in the case of CPADB) with the amine groups at MSN-NH_2_ surface. In this case, the nanoparticle acts as a leaving R group (Figure 4). The propagating radicals are thus located on the terminal end of the surface-grafted polymer, facilitating the growth of the polymer chains. This process has advantages over the Z-group approach, where the RAFT agent is immobilized by the Z group and the propagating radicals are in solution (Figure 4). In the Z-group approach the chain-transfer reactions between the propagating polymer radicals and the anchored CTA occur near the surface and are hampered by the steric hindrance from the neighboring attached polymer chains and the low availability of the chain end-group [30].

The gRAFT-*from* polymerizations were performed with MSN-CTA using different monomer concentrations and CTA:initiator ratios. The amount of polymer in the shell was determined by ^1^H NMR (Table 2). The expected molecular weight for 100% conversion is *M*_n_ ≈ 50 kDa. The actual molecular weight of the MSN-grafted polymer was not determined because recovery of the polymer by cleavage of the chain-MSN bond have very low yields after purification. This is one of the disadvantages of gRAFT-*from*, relative to the gRAFT-*to* approach.

Using a 1:10 initiator:CTA ratio we obtained 0.8 and 2.8 wt % of polymer shell (polymer relative to silica weight), for reactions with 0.5 and 2 M of monomer (Table 2). These results were expected, since a larger monomer concentration leads to a longer polymer chains. The initiator:CTA ratio is related to the number of chains initiated at the particles surfaces (CTA) and in solution (initiator). By increasing the relative number of solution-initiated chains (to a 1:5 initiator:CTA ratio), the amount of polymer incorporated in the MSNs decreases to approximately half (Table 2).

Overall, although the gRAFT-*from* process yields higher polymer incorporation than gRAFT-*to*, the amounts obtained are still limited (even taking into account the very low density of MSNs). The difficulty in increasing these values is probably related with the increased termination reactions between chains growing from the surface as the grafting density is increased (gRAFT-*from*). Furthermore, as the polymer chains grow, there is an increase in the steric hindrance for monomer units to diffuse to the particle surface and polymerize (gRAFT-*to*) [40].

### 3.3. Crosslink-grafting Approach (gRAFT-cross)

Since the gRAFT-*from* approach provides higher surface coverage, we used this method to improve polymer incorporation. Our first modification was to introduce a cross-linking agent to reduce the growing polymer chain mobility and thus decreased the termination reactions between chains, in a crosslink-grafting approach (gRAFT-*cross*). To test this hypothesis, we replicate the previous polymerization reactions (Table 2) in the presence of ethylene glycol dimethacrylate (EGDMA) as crosslinker (Table 3). The results confirm that by cross-linking the polymer the efficiency of the gRAFT-*from* method is improved, resulting in a ca. two-fold increase on the amount of polymer incorporated for the same conditions. The best incorporation results were obtained when the initiator:CTA ratio and the monomer:initiator ratio were higher (initiator:CTA = 1:10 and [DAEM] = 2.0 M).

### 3.4. Hybrid Grafting Approach (gRAFT-hybrid)

Our second approach to improve polymer incorporation is based on previous reports that the addition of free CTA in the polymerization medium containing CTA-modified nanoparticles improves the control of the polymerization reaction at the surface, leading to higher values of grafted polymer [41]. We have improve this procedure by developing a new hybrid grafting method (gRAFT-*hybrid*), where the MSN-CTA are added to a reaction medium with both monomer and free-growing RAFT polymer chains [2]. The MSN-CTA were added before the conversion plateau of the free-growing chains (at approximately 50% conversion) (Figure S1, Supporting Information). Due to the higher concentration of CTA in the MSN surface (two times relative to that in the free polymer chains) the remaining monomer start to react with the CTA-MSN, initiating a gRAFT-*from* process. As the monomer concentration decreases, termination reactions between free and MSN-grafted chains increase polymer incorporation, although at the expected cost of a broader chain size distribution. Characterization of the resulting polymer is however difficult, as usual for the grafting-from process.

The polymerization reaction was performed using different monomer:CTA ratios and the polymer incorporation was determined by ^1^H NMR (Table 4). The target molecular weight for the free chains at ≈50% conversion (when the MSN-CTA are added to the polymerization medium) was 4 and 30 kDa, for MSN-pDAEM-*hybrid*-1 and MSN-pDAEM-*hybrid*-2, respectively.

Our results confirm that this approach results in a higher polymer incorporation than the obtained by gRAFT-*from* (Table 1) and gRAFT-*to* methods (Table 2). Since the main difference to the original process are the free polymer chains in solution, we can conclude that their presence and consequent termination reactions with the chains growing from the surface are crucial to obtain a higher amount of incorporated polymer [41]. This is confirmed by the larger amount of polymer incorporated in the MSN shell when higher molecular weight free growing chains are used.

### 3.5. RAFT-based Grafting for Polymer Incorporation onto Nanocarriers

Optimization of the amount of polymer that is incorporated in the surface of the MSNs of hybrid nanocarriers is of paramount importance to avoid cargo leakage on the non-release state [1,2]. We have previously shown that a polymer shell with approximately 5 wt % of polymer offers the better performance in similar smart nanocarriers for controlled cargo release [2]. In fact, lower polymer amounts led to residual release in the OFF (cargo retention) state, while larger polymer amounts impact the release performance of the system by increasing cargo retention in the ON (cargo release) state.

The two more common RAFT grafting methods, gRAFT-*to* and gRAFT-*from*, offer limited polymer incorporation (even considering the very low density of MSNs), with the maximum amount of polymer obtained for gRAFT-*to* (1 wt %) being lower than that obtained by gRAFT-*from* (2.8 wt %), both below the target 5 wt % polymer content. The tested modifications to the gRAFT-*from* approach effectively improved the incorporation of polymer in the nanocarriers. Both the use of a crosslinking agent (gRAFT-*cross*) and the introduction of free-growing RAFT polymer chains in the reaction medium (gRAFT-*hybrid*), resulted in polymer incorporations above 5 wt %. The larger amounts of polymer shell were obtained for gRAFT-*cross* when the initiator:CTA ratio and the monomer concentration were higher (same conditions for which gRAFT-*from* had higher yield) and for gRAFT-*hybrid* when the added free growing polymer chains had larger molecular weight (same conditions for which gRAFT-*to* had higher yield). 

In both gRAFT-*to* and gRAFT-*from*, the grafting yield is limited by the steric hindrance issues. In gRAFT-*to*, the surface coverage is low due to steric hindrance between chains that are already anchored to the surface and those approaching it. Also, the diffusion of the polymer chain end-group to the surface is hindered by the chain conformation. Although both effects lead to a lower coverage as the polymer chain size increases, the amount of incorporated polymer is determined by the balance between the surface coverage achieved and the molecular weight of the polymer. In our case, it increases from 0.5 to 1.0 wt % when the *M_n_* increase from 15 to 47 kDa. On the other hand, the gRAFT-*to* approach provides excellent control over the size, composition and architecture of the polymer chains and also allows complete characterization of the polymer chains before incorporation. In the case of gRAFT-*from* it is possible to reach higher coverage because the chains grow from the surface, initially with very low steric hinderance. However, as the oligoradicals grow in size, chain termination rates can increase due to radical vicinity. The obtained polymer incorporation was however higher than that obtained with gRAFT-*to*.

The larger polymer incorporation of gRAFT-*cross* compared to the gRAFT-*from* approach (for the same experimental conditions) is probably due to the fact that the growing polymer has a more constrained conformation, decreasing the termination rate and probably improving the accessibility of the monomer units. This resulted in a maximum two-fold increase (to ca. 5 wt %) on the amount of grafted polymer, relative to gRAFT-*from*.

The introduction of free-growing RAFT polymer chains in the gRAFT- *hybrid* approach, also resulted in an increase on the amount of grafted polymer to a maximum of ca. 5 wt %. This value corresponds to around two-fold increase on the amount of polymer incorporation relative to the best result of gRAFT-*from* and a five-fold increase relative to gRAFT-*to*. In the uncommon mechanism of gRAFT-*hybrid*, the crucial factor is the simultaneous growing of free chains and MSN-grafted chains. The free growing chains eventually are involved in termination reactions with the surface-bound growing chains, resulting in the observed increase in the amount of incorporated polymer, although at the expense of less homogeneous chain size distribution in the particle shell.

## 4. Conclusions

The amount of stimuli-responsive polymer on the shell of nanocarriers is an important parameter that determines the performance of controlled release by the system [1,2]. Here, we prepared a nanocarrier for pH-actuated controlled release, with a mesoporous silica core and a pH-responsive polymer shell prepared by RAFT polymerization. To optimize the amount of polymer that is incorporated in the MSN, we tested different grafting strategies. The two more common RAFT grafting methods, gRAFT-*to* and gRAFT-*from*, result in limited amounts of polymer incorporation onto the MSN surface, mainly due to steric hindrance impairing the chain-surface reaction in the first case and increased chain termination rates (due to radical proximity) in the second case. However, although gRAFT-*to* leads to comparably lower coverages, it allows characterization of the polymer chains before incorporation, providing better control of the shell composition.

We were able to considerably increase the amount of polymer incorporation with two modifications of the gRAFT-*from* approach, either using a crosslinking agent to constrain chain conformation, decreasing the termination rate and improving the accessibility to the reacting monomer (gRAFT-*cross*) or introducing free-growing RAFT polymer chains that react with surface growing chains to increase polymer incorporation, although with lower size homogeneity (gRAFT-*hybrid*).

## Figures and Tables

**Table 1:** Molecular weight of polymers obtained by RAFT (M_n_ values by SEC-MALS) and weight fraction of polymer in the MSN shell incorporated by the grafting-*to* approach (gRAFT-*to*).

	$M_{n}^{SEC}$ (kDa)	$M_{n}^{Theo}$(kDa)	Polymer Incorporation (wt % MSN)
**MSN-pDAEM15-*to***	15	9	0.5
**MSN-pDAEM47-*to***	47	44	1.0

**Table 2:** Weight fraction of pDAEM in the MSN shell incorporated by grafting-*from* approach.

[DPAEM] (M)	Initiator:CTA Ratio	
	1:5	1:10
**0.5**	0.4 %	0.8 %
**1.3**	0.5 %	-
**2.0**	0.8 %	2.8 %

**Table 3:** Weight fraction of polymer in the MSN shell incorporated by grafting-from the nanoparticles in the presence of cross-linking agent ethylene glycol dimethacrylate (EGDMA) (gRAFT-*cross*).

**[DPAEM] (M)**	**Initiator:CTA Ratio**	
	**1:5**	**1:10**
**0.5**	0.5%	2.1%
**1.3**	1.6%	-
**2.0**	1.9%	5.1%

**Table 4:** Weight fraction of polymer in the MSN shell incorporated by hybrid grafting-to the nanoparticles (gRAFT-*hybrid*).

Polymer Incorporation (MSN, wt %)	
MSN-pDAEM-*hybrid*-1	1.4
MSN-pDAEM-*hybrid*-2	5.2

## Supplementary Materials

The following are available online at https://www.mdpi.com/2073-4360/12/10/2175/s1, Figure S1. Reaction conversion of DAEM followed by the disappearance of the vinylic protons in the ^1^H NMR. Figure S2. ^1^H-NMR of the polymerization of DAEM in CDCl_3_, at several time points. The consumption of DAEM is visible in the disappearance with time of the vinylic protons (5.56 ppm and 6.10 ppm). The formation of polymer is evident with the shifting of the peak corresponding to the ether protons (4.07 to 3.87 ppm) with time. Trioxane (5.17 ppm, C_3_H_6_O) was used as internal standard. Figure S3. Solution ^1^H-NMR of MSN-NH_2_ (at pH = 13) in D_2_O, with peaks assigned for the APTES propyl chain, showing surface modification of the nanoparticles. Residual ethanol protons are denoted by (*). Figure S4. The amount of RAFT agent at the MSNs surface was calculated by subtracting the light scattering contribution (measured for bare MSNs, grey curve), from the absorption spectrum of MSN-CTA (blue curve) at the of absorption maximum wavelength (305 nm) and using the molar extinction coefficient of the RAFT. Figure S5. GPC/SEC chromatograms of pDAEM15 (A) and pDAEM47 (B). Refractive index (RI) signal (red) and multiangle light scattering (MALS) signal at 90° (black) vs. elution volume. Molecular weight calculated for each elution volume (blue dots).
