# Supplementary material for: Grafting with RAFT—gRAFT Strategies to Prepare Hybrid Nanocarriers with Core-shell Architecture
Source: Polymers (Basel). 2020 Sep 23;12(10):2175. doi: 10.3390/polym12102175 (PMC7598713; doi:10.3390/polym12102175)
Supplement: Supplementary file 1 [file polymers-12-02175-s001.pdf]

Supporting Information

Grafting with RAFT: gRAFT strategies to prepare hybrid nanocarriers with core-shell architecture

José L. M. Gonçalves, Edgar J. Castanheira, Sérgio P. C. Alves, Carlos Baleizão and José Paulo S. Farinha**

Centro de Química Estrutural and Department of Chemical Engineering, Instituto Superior Técnico, Universidade de Lisboa, 1049-001 Lisboa, Portugal

E-mail: carlos.baleizao@tecnico.ulisboa.pt (C.B.); farinha@tecnico.ulisboa.pt (J.P.S.F.)

Keywords: core-shell hybrid mesoporous silica nanoparticles; pH-responsive polymers; controlled release; RAFT

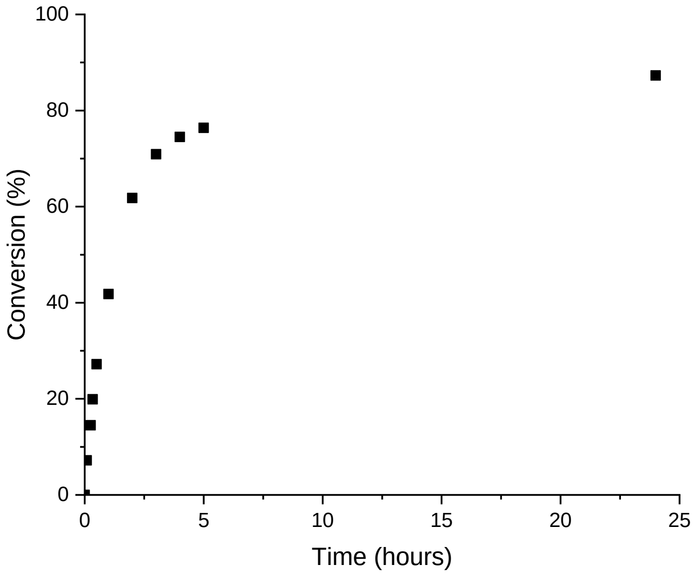

Figure S1. Reaction conversion of DAEM followed by the disappearance of the vinylic protons in the ^{1}H NMR.

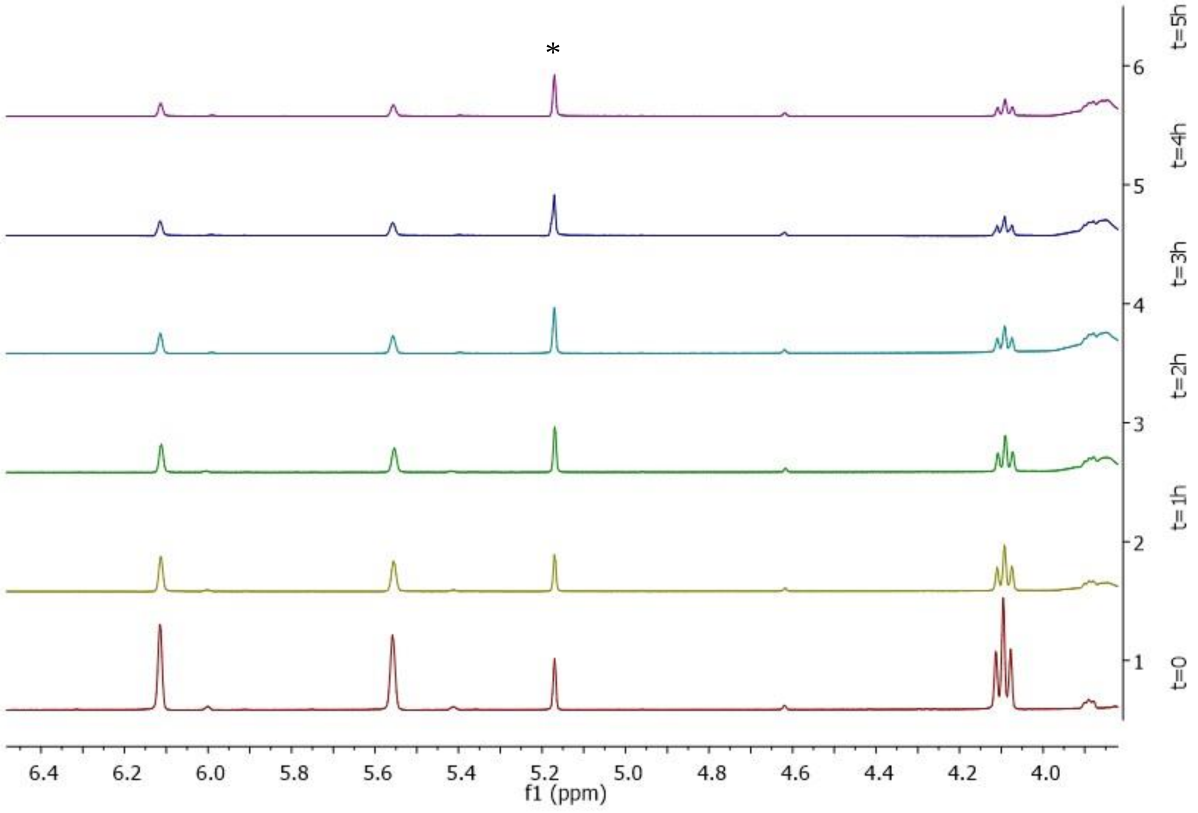

Figure S2. ^{1}H-NMR of the polymerization of DAEM in $CDCl_3$, at several time points. The consumption of DAEM is visible in the disappearance with time of the vinylic protons (5.56 ppm and 6.10 ppm). The formation of polymer is evident with the shifting of the peak corresponding to the esther protons (4.07 to 3.87 ppm) with time. Trioxane (5.17 ppm, C_3H_6O) was used as internal standard.

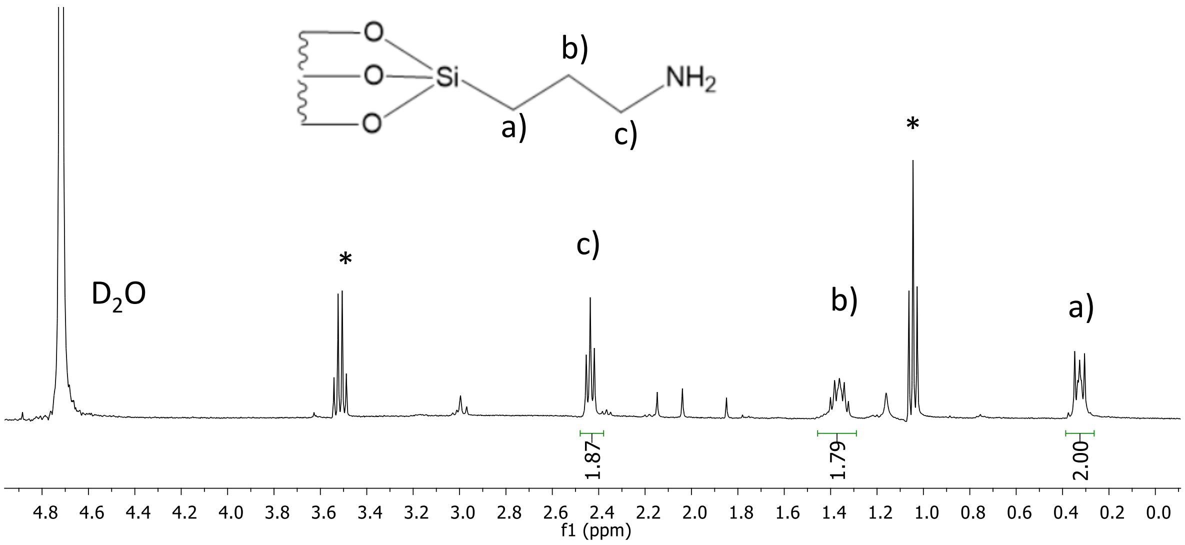

Figure S3. Solution ^{1}H-NMR of MSN-NH_2 (at pH = 13) in D_2O, with peaks assigned for the APTES propyl chain, showing surface modification of the nanoparticles. Residual ethanol protons are denoted by (*).

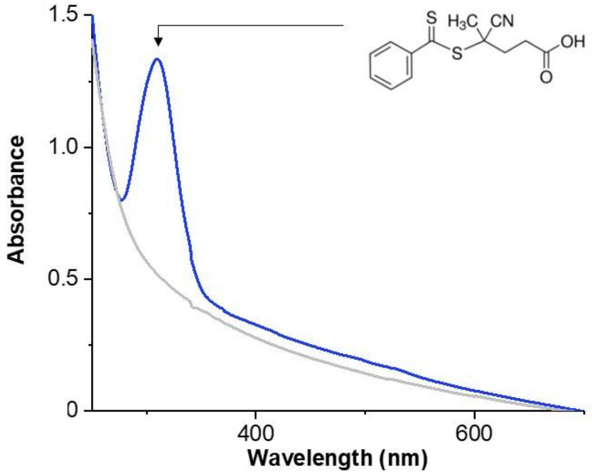

Figure S4. The amount of RAFT agent at the MSNs surface was calculated by subtracting the light scattering contribution (measured for bare MSNs, grey curve), from the absorption spectrum of MSN-CTA (blue curve) at the of absorption maximum wavelength (305nm), and using the molar extinction coefficient of the RAFT.

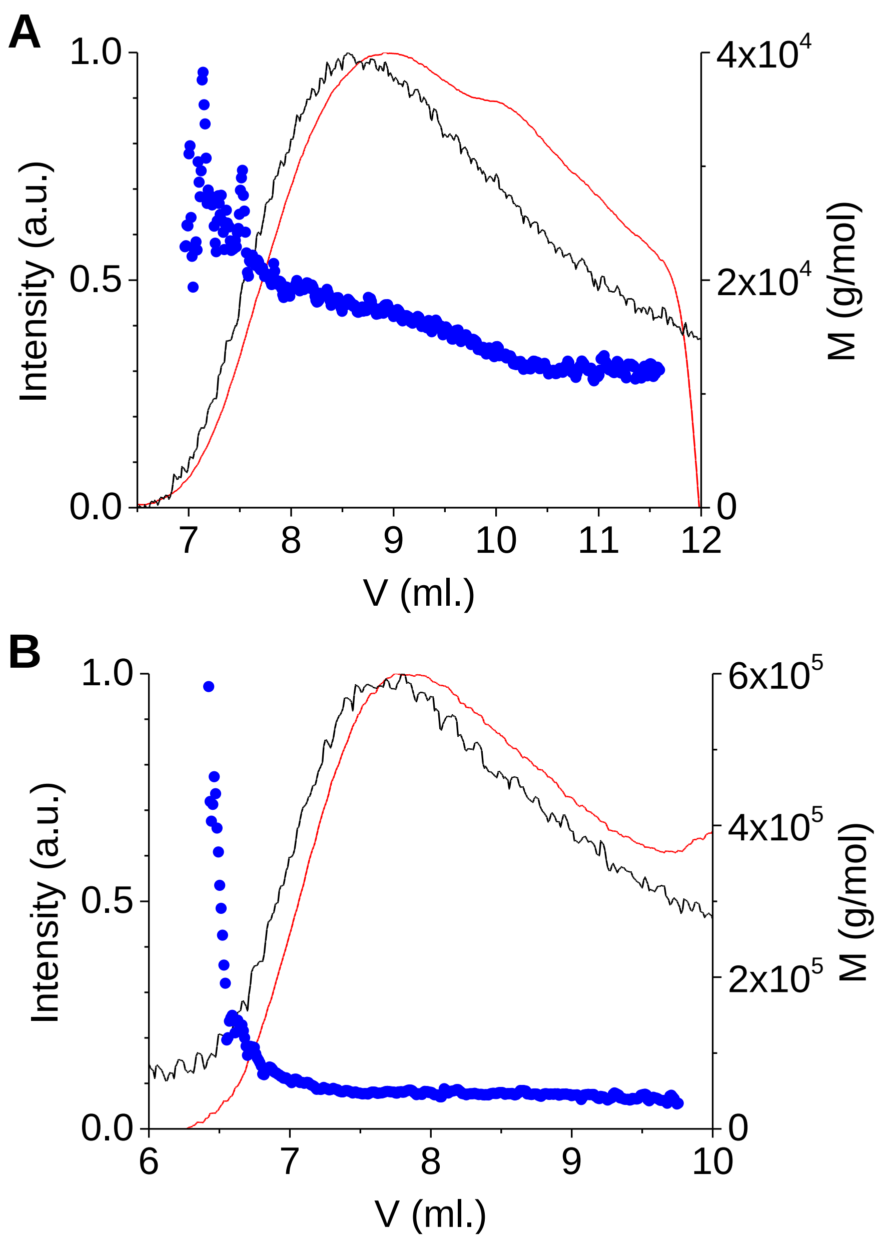

Figure S5. GPC/SEC chromatograms of pDAEM15 (A) and pDAEM47 (B). Refractive index (RI) signal (red) and multiangle light scattering (MALS) signal at 90° (black) vs. elution volume. Molecular weight calculated for each elution volume (blue dots).
